# Honokiol inhibits bladder tumor growth by suppressing EZH2/miR-143 axis

**DOI:** 10.18632/oncotarget.6135

**Published:** 2015-10-15

**Authors:** Qing Zhang, Wei Zhao, Changxiao Ye, Junlong Zhuang, Cunjie Chang, Yuying Li, Xiaojing Huang, Lan Shen, Yan Li, Yangyan Cui, Jiannan Song, Bing Shen, Isaac Eliaz, Ruimin Huang, Hao Ying, Hongqian Guo, Jun Yan

**Affiliations:** ^1^ Department of Urology, Drum Tower Hospital, Medical School of Nanjing University; Institute of Urology, Nanjing University, Nanjing, Jiangsu, China; ^2^ State Key Laboratory of Pharmaceutical Biotechnology and MOE Key Laboratory of Model Animals for Disease Study, Model Animal Research Center of Nanjing University, Nanjing, Jiangsu, China; ^3^ Key Laboratory of Food Safety Research, Institute for Nutritional Sciences, Shanghai Institutes for Biological Sciences, University of Chinese Academy of Sciences, Chinese Academy of Sciences, Shanghai, China; ^4^ Department of Urology, Shanghai General Hospital, Shanghai Jiaotong University, Shanghai, China; ^5^ Amitabha Medical Clinic and Healing Center, Santa Rosa, CA, USA; ^6^ SIBS (Institute of Health Sciences)-Changhai Hospital Joint Center for Translational Research, Institutes for Translational Research (CAS-SMMU), Shanghai, China

**Keywords:** honokiol, bladder cancer, EZH2, microRNA

## Abstract

The oncoprotein EZH2, as a histone H3K27 methyltransferase, is frequently overexpressed in various cancer types. However, the mechanisms underlying its role in urinary bladder cancer (UBC) cells have not yet fully understood. Herein, we reported that honokiol, a biologically active biphenolic compound isolated from the *Magnolia officinalis* inhibited human UBC cell proliferation, survival, cancer stemness, migration, and invasion, through downregulation of EZH2 expression level, along with the reductions of MMP9, CD44, Sox2 and the induction of tumor suppressor miR-143. Either EZH2 overexpression or miR-143 inhibition could partially reverse honokiol-induced cell growth arrest and impaired clonogenicity. Importantly, it was first revealed that EZH2 could directly bind to the transcriptional regulatory region of miR-143 and repress its expression. Furthermore, honokiol treatment on T24 tumor xenografts confirmed its anticancer effects *in vivo*, including suppression tumor growth and tumor stemness, accompanied by the dysregulation of EZH2 and miR-143 expressions. Our data suggest a promising therapeutic option to develop drugs targeting EZH2/miR-143 axis, such as honokiol, for bladder cancer treatment.

## INTRODUCTION

Urinary bladder cancer (UBC) is one of the most common urogenital malignant tumors, with an estimated 74,690 new cases and 15,580 deaths occurring in USA during 2014 [1]. Although localized bladder cancers can be surgically resected, the recurrence and progression rates are extremely high [2]. Moreover, the therapeutic response from the patients with advanced bladder cancer with radio- or chemotherapy is very limited. Therefore, UBC is one of the most expensive malignancies, with the annual cost at 4 billion US$ for UBC in the USA per year [3, 4].

Recent studies revealed that a small population of cancer stem cells (CSCs), which is enriched after therapy, may account for chemotherapy failure [5-7]. These CSCs have the ability to generate all types of differentiated cells to repopulate tumors and eventually lead to metastasis; they are thus regarded as the sustaining force of a tumor. The CSC-targeting therapeutic approach to prevent tumor recurrence becomes a promising regimen for UBC treatment.

Histone modification through polycomb repressive complexes (PRCs) plays an essential role for normal and malignant cell stemness maintenance [8, 9]. As a pivotal component of PRC2, Enhancer of Zeste Homologue 2 (EZH2) has intrinsic histone methyltransferase activity for histone H3K27. The increased trimethylation on H3K27 by EZH2 activation specifically represses the transcription of differentiation-related genes throughout the cell cycle to maintain the stemness of cells [10, 11]. EZH2 deregulation is frequently detected in a variety of cancer types, including lung cancer, prostate cancer and UBC [11-13], by regulating its multiple target genes involved in carcinogenesis.

Recent studies revealed that microRNAs (miRNAs) as a new class of regulators for tumor development. miRNAs belong to the noncoding RNAs with approximately 22 nucleotides, functioning as negative regulators for gene expression at the post-transcriptional level. Previous studies indicated that miR-101 enhanced cytostatic drug sensitivity of liver cancer through targeting EZH2 gene [14, 15]. In reverse, several tumor suppressor miRNAs and proapoptotic miRNAs have been identified as the direct targets of EZH2 [16, 17]. Exploring the EZH2-miRNAs-associated signaling network in UBC development and identification of new EZH2 inhibitors may provide novel therapeutic strategies for UBC treatment.

Honokiol is one of the lignans with high bioavailability, which can be extracted from *Magnolia officinalis*. It has been long to be used in traditional Chinese medicine, showing multiple biological activities, including anticancer, anti-inflammation, and neuroprotective properties [18, 19]. However, the molecular mechanism of anti-cancer effects of honokiol on bladder cancer cells remains unclear.

In this study, we have determined the effects of honokiol through the repression of oncoprotein EZH2 and induction of tumor suppressor miR-143, on the UBC cell proliferation, survival, cancer stemness maintenance and cell migration *in vitro* and *in vivo*. Furthermore, the novel mechanism how EZH2 directly regulates miR-143 in UBC cells has also been uncovered.

## RESULTS

### The anticancer effect of honokiol *in vitro*

**Figure 1:**
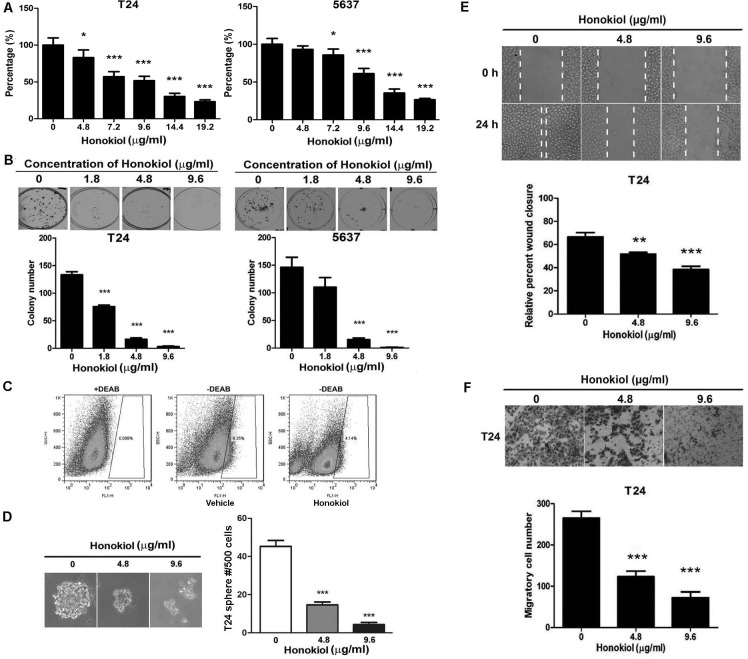
Honokiol decreased the aggressiveness of UBC cells Cell survival **A.** and colony formation capacities **B.** of T24 and 5637 cells treated with honokiol at the indicated concentrations. **C.**, ALDH^High^ cell population (stem and progenitor cells) assessed by flow cytometric analysis in T24 cells treated with 9.6 μg/ml honokiol for 24 h. Diethylaminobenzaldehyde (DEAB), an ALDH-specific inhibitor, was used as a negative control. **D.**, Tumor sphere formation capacity of T24 cells with honokiol at the indicated concentrations. Lower panel, representative photographs of spheres under honokiol treatment. **E.**, Cell migration capacity examined by the wound healing assay. Relative wound closure percentage at 24 h was normalized by the gap distance at 0 h. Vertical dotted lines indicate the wound formed at 0 h (top row) and 24 h (bottom row). **F.**, Cell invasive capacity was detected by the Transwell assay. Representative photograph of the transwell inserts with invasive cells at 20× magnification. All data are represented as mean ± SEM from triplicate wells. *, *P* < 0.05, **, *P* < 0.01, ***, *P* < 0.001, compared with the vehicle-treated group indicated as “0”.

The anticancer effect of honokiol on human bladder cancer cells was investigated in three commonly used human UBC cell lines, T24, 5637 and J82. Different concentrations of honokiol ranging from 4.8 to 19.2 μg/ml were applied to UBC cells. After 72 h treatment, cell viability was assessed by MTT assay showing that honokiol reduced viable cell number in a dose-dependent manner (Figure 1A and Supplementary Figure 1A). The lowest honokiol concentrations for significant growth inhibition of T24, 5637 and J82 cells are 4.8, 7.2 and 4.8 μg/ml, respectively. Since the cytotoxic effects are similar among three UBC cell lines, we chose two commonly used human UBC cell lines, T24 and 5637, for further experiments. Colony formation assay was also performed to test the long-term effects of honokiol. As shown in Figure 1B, clonogenicities of T24 and 5637 cells were dramatically decreased upon long-term honokiol treatment with a concentration higher than 4.8 μg/ml.

CSC is an underlying cause of tumor recurrence and metastasis, and it is becoming the primary target for cancer treatment. Here, whether honokiol could inhibit the stemness of UBC cells was investigated. ALDEFLUOR assay was utilized to identify the stem/progenitor cells based on the high expression level of aldehyde dehydrogenase (ALDH) enzyme. The treatment of honokiol for 24 h reduced the ALDH^High^ cell population in T24 (4.13%) and 5637 cells (3.39%), comparing with that in the vehicle-treated T24 (8.34%) and 5637 (8.69%) cells (Figure 1C and Supplementary Figure 2A). Consistently, sphere formation capacity of T24 cells was also inhibited by honokiol for 14 d treatment (Figure 1D). The number of spheres in honokiol-treated cells (4.8 and 9.6 μg/ml) was 64.4% and 88.9% less than that in vehicle-treated cells, respectively.

Cell migration by wound healing assay and cell invasion by Transwell assay were further performed. Honokiol treatment in T24 and 5637 cells delayed wound closure in a dose-dependent manner (Figure 1E and Supplementary Figure 2B). In the presence of 4.8 μg/ml honokiol, the invasive capacities of T24 and 5637 cells were also significantly decreased by 55.6% and 83.4%, compared to vehicle treatment. Such inhibitions were enhanced at 9.6 μg/ml honokiol, *i.e.* 72.2% reduction in T24 and 90.7% in 5637 cells (Figure 1F and Supplementary Figure 2C).

To dissect the anticancer effects, we found honokiol induced G1 arrest and the increase of Sub-G1 population (apoptotic cells) in a concentration-dependent manner (Supplementary Figure 3A). Western blotting assay further revealed that cell proliferation protein (Cyclin D1) was reduced and cell cycle inhibitors (p21 and p27) were increased (Supplementary Figure 3B). In addition, honokiol treatment reduced the expression level of anti-apoptotic protein (Bcl2) and increased that of pro-apoptotic protein (BAX), accompanied by the cleavages of PARP (Supplementary Figure 3C). Taken together, these results demonstrated that honokiol inhibits the cell proliferation, survival, stemness and invasion of bladder cancer cells *in vitro*.

### The anticancer effect of honokiol is through repressing EZH2

**Figure 2:**
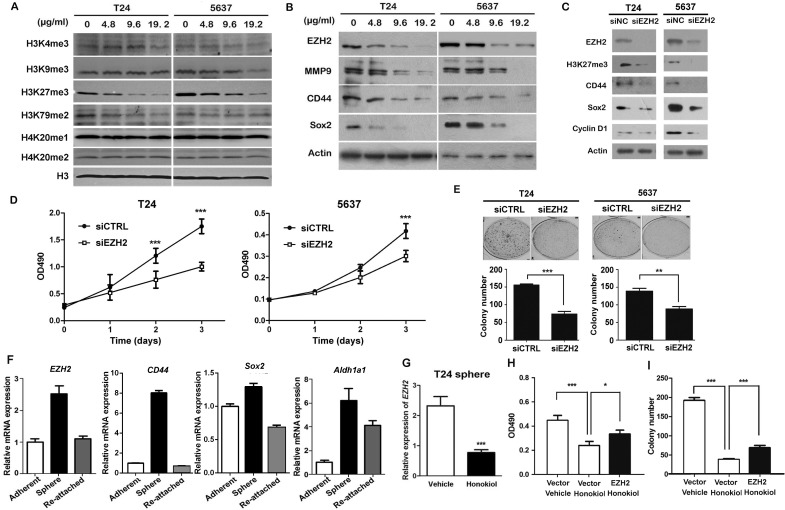
Honokiol suppressed the expression of EZH2 gene **A.**, Detection of methylation changes of histones under the treatment of honokiol in both T24 and 5637 cells for 24 h. Histone H3 was used as the loading control. **B.**, The expression levels of EZH2, MMP9, CD44 and Sox2 proteins in T24 and 5637 bladder cancer cells treated with 9.6 μg/ml honokiol for 24 h by Western blotting assay. β**-**actin protein was used as the loading control. **C.**, The expression levels of EZH2, H3K27me3, CD44, Sox2, and Cyclin D1 proteins in T24 and 5637 bladder cancer cells with EZH2 knockdown. **D.**, Cell proliferation of T24 and 5637 cells with EZH2 knockdown by small interfering RNA (siEZH2) for 3 days by MTT assay. **E.**, Colony formation capacities of T24 and 5637 cells with EZH2 knockdown. **F.**, EZH2 mRNA level in T24 tumor sphere (stem/progenitor cell population) and T24 cells re-attached on cell plates (differentiated cell population) by quantitative RT-PCR. EZH2 mRNA level in T24 parental cells under regular culture condition (adherent) was used as the normalization control. **G.**, Relative EZH2 mRNA level in T24 tumor sphere treated with 9.6 μg/ml honokiol by qRT-PCR. β**-**actin gene was used as the normalization control. Ectopic overexpression of EZH2 gene reversed the honokiol-induced cytotoxicity by MTT assay **H.** and clonogenicity **I.** in T24 cells. The empty backbone plasmid (pcDNA3) was used as the negative control (Vector). *, *P* < 0.05; **, *P* < 0.01; ***, *P* < 0.001.

Since epigenetic modifications are involved in cell survival, invasion, and stemness maintenance, we examined the global changes in several histone marks in honokiol-treated UBC cells. Among these histone marks, we observed that honokiol selectively and strikingly reduced the trimethylation of histone 3 lysine 27 (H3K27me3) in T24, 5637 and J82 cells (Figure 2A and Supplementary Figure 1B). Besides, the reductions of H3K4me3 and H3K79me2 were detected in either 5637 or T24 cells (Figure 2A). Taken together, honokiol can reduce H3K27me3 level, independent on UBC cell lines.

EZH2 is the H3K27 methyltransferase, which is overexpressed in various cancer types, including bladder cancer. Therefore, we first confirmed that all 7 UBC cell lines express EZH2, with relatively lower levels in T24 and J82 cells and a higher level in 5637 cells (Supplementary Figure 1C). Honokiol decreased the expression level of EZH2 protein in a dose-dependent manner in T24, J82 and 5637 cells, regardless of their different expression levels of EZH2 (Figure 2B and Supplementary Figure 1B). The decrease of EZH2 in T24 and 5637 cells was accompanied by the reduction of MMP9 (invasion-associated marker), and other stem cell markers (including CD44 and Sox2; Figure 2B).

In order to test the role of EZH2 in bladder cancer cells, we knocked down EZH2 by small interfering RNA and found its depletion reduced cell proliferation by 33.9% and 27.9%, 72 h after siRNA transfection (Figure 2C and 2D). Consistently, colony formation capabilities were also reduced by 52.6% and 36.5% in RNAi group, compared to control siRNA group (Figure 2E). EZH2 ablation reduced trimethylated H3K27, Cyclin D1 and the expression of stem cell markers (CD44 and Sox2) in T24 and 5637 cells, respectively (Figure 2C). A tumor sphere model, in which the stem cells have the capability to form the spheres under the suspension culture condition, was then generated. Once the stem cells dissociate from the spheres and re-attach the plates, they lose their stemness and undergo differentiation. Using this model, we showed EZH2 mRNA level increased 2.52 folds in T24 spheres, compared to adherent T24 cells. Interestingly, EZH2 expression in re-attached cells reduced to comparable level (1.10-fold) with the T24 cells under regular culture condition. We further confirmed that the expression levels of EZH2 and stem cell markers (*CD44, Sox2*, *Aldh1a1*, *Pou5f1*, and *Nanog*) were also associated with the cell differentiation status (Figure 2F and supplementary Figure 4). These data demonstrated EZH2 could regulate the cell proliferation and stemness of bladder cancer cells *in vitro*.

EZH2 mRNA level in T24 tumorsphere was downregulated by 65.4% with honokiol treatment for 24 h (Figure 2G). On the other hand, ectopic expression of EZH2 in T24 cells could partially reverse the honokiol-induced inhibitions of cell growth (Figure 2H) and clonogenicity (Figure 2I). These findings indicate the honokiol-induced anti-cancer effects are partially through downregulation of EZH2 gene, a crucial regulator of cell proliferation and stemness.

### The anticancer effect of honokiol is through induction of miR-143

**Figure 3:**
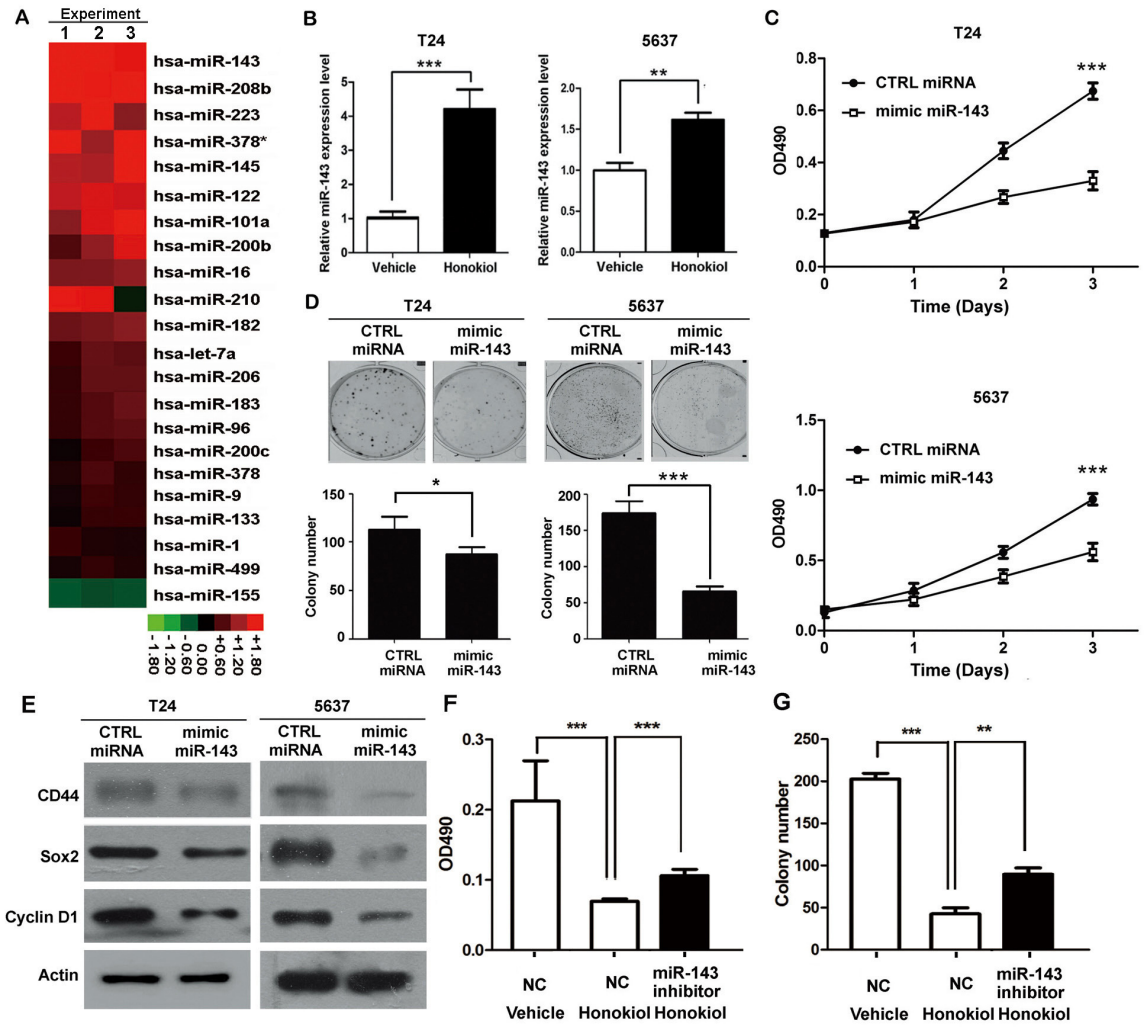
Honokiol induced the expression of miR-143 **A.**, Expression levels of 22 cancer-related miRNAs in honokiol-treated T24 cells by qRT-PCR, normalized by the vehicle-treated group. The normalized values were shown in the heat map in Red-Black-Green color scheme. **B.**, Relative expression levels of miR-143 in T24 and 5637 cells treated with honokiol (9.6 μg/ml) by qRT-PCR. U6 snRNA was used as the normalization control. **C.**, Cell proliferation of T24 and 5637 cells with ectopic expression of mimic miR-143 for 3 days by MTT assay. CTRL miRNA, the non-targeting miRNA control. **D.**, Colony formation capacity of T24 and 5637 cells with miR-143 overexpression. **E.**, The expression levels of CD44, Sox2, and Cyclin D1 proteins in T24 and 5637 bladder cancer cells with miR-143 overexpression. **F.** & **G.**, miR-143 inhibitor reversed the honokiol-induced cytotoxicity by MTT assay **F.** and clonogenicity **G.** in T24 cells. NC, Anti-miR negative control. *, *P* < 0.05; **, *P* < 0.01; ***, *P* < 0.001, as compared to the control group.

miRNAs have been implicated in various cellular processes, including drug response. Therefore, we carried out a small scale qRT-PCR screening in 22 cancer-related miRNAs to identify the miRNAs responsible for honokiol treatment. Honokiol at 9.6 μg/ml in T24 cells induced 8 miRNAs upregulated (≥2.0-folds, Figure 3A). Among them, miR-143 is the one with the highest change. The upregulation of miR-143 upon honokiol treatment was further verified in 5637 and J82 cells (Figure 3B and Supplementary Figure 1D). As a negative control miRNA, miR-1 was not significantly changed in both of two cell lines (Supplementary Figure 5). miR-34a, whose upregulation was reported very recently by honokiol in breast cancer cells [20], was used as a positive control miRNA. Up-regulation of miR-34a upon honokiol treatment was also identified in both UBC cell lines, T24 and 5637 (Supplementary Figure 5).

In order to test whether miR-143 is essential for bladder cancer cell proliferation and stemness, miRNA mimic for miR-143 (mimic miR-143) was transiently transfected into T24 and 5637 cells. Mimic miR-143 significantly inhibited UBC cell proliferation (Figure 3C) and clonogenicity (Figure 3D). Consistently, mimic miR-143 reduced Cyclin D1 protein level, compared to control group (Figure 3E). In addition, the protein levels of CSC markers, such as CD44 and Sox2, were also decreased with the miR-143 mimic transfection (Figure 3E).

Whether miR-143 is required for honokiol-induced anti-cancer effects was studied using the miRNA inhibitor for miR-143 (miR-143 inhibitor. A random sequence anti-miR molecule with an undetectable effect on known miRNA function was used as an anti-miR negative control (NC). Under 9.6 μg/ml honokiol treatment, miR-143 inhibitor could partially restore the honokiol-induced cell growth arrest (Figure 3F) and reduction of colony number (Figure 3G) in T24 cells. Hence, these data reveal that miR-143 overexpression, induced by honokiol treatment, could suppress cell proliferation and stemness maintenance.

### EZH2 directly binds to miR-143 promoter to repress its expression

**Figure 4:**
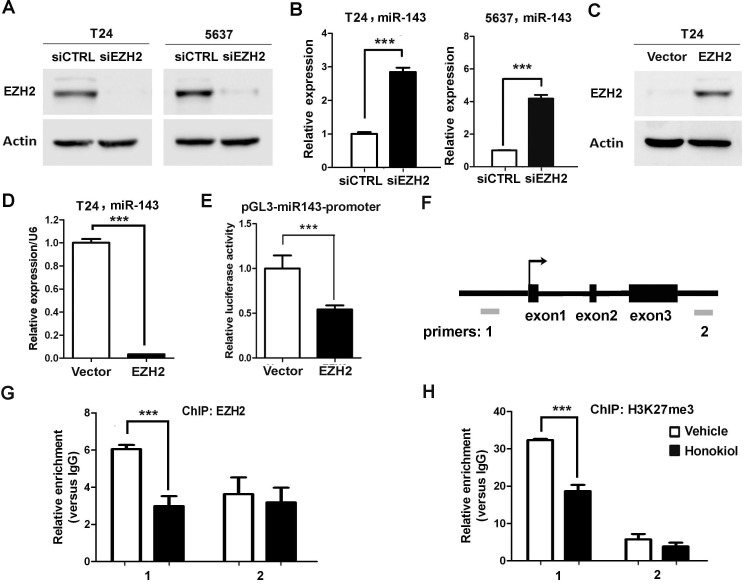
EZH2 directly regulated miR-143 expression in UBC cells **A.**, Western blotting data showed the knockdown effects of EZH2 in T24 and 5637 cells. siCTRL is the non-targeting and normalization control. **B.**, Expression levels of miR-143 by EZH2 knockdown (siEZH2) in T24 and 5637 cells, detected by qRT-PCR. **C.**, Western blotting data showed the overexpression of EZH2 in T24 cells. **D.**, Overexpression of EZH2 suppressed miR-143 expression by qRT-PCR. **E.**, Luciferase assay showed repression of transcriptional activity of the miR-143 promoter. **F.**, A schematic representation of miR-143 genomic structure. Grey bars, amplicons in the ChIP assay. **G.** and **H.**, The recruitment of EZH2 **G.** and the trimethylation level of H3K27 **H.** on miR-143 locus upon 9.6 μg/ml honokiol treatment in T24 cells for 24 h. Normal rabbit IgG was used as the negative control of ChIP assay. **, *P* <0.01; ***, *P* <0.001, as compared to the control group.

**Figure 5:**
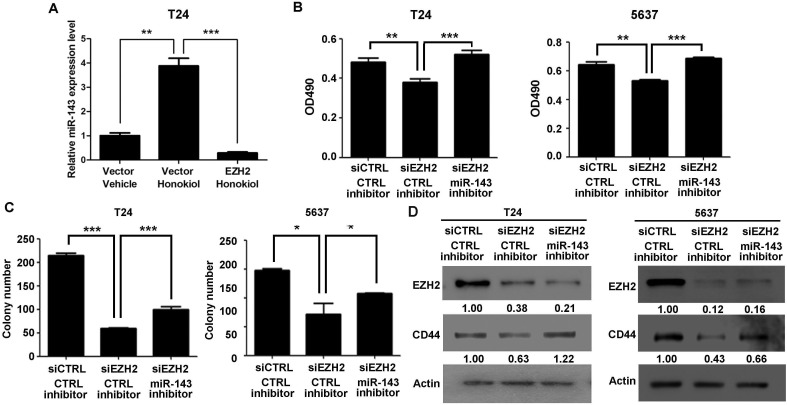
EZH2 gene regulated cell proliferation and clonogenicity through miR-143 in UBC cells **A.** T24 cells were transfected with EZH2 expressing plasmid, followed by 9.6 μg/ml honokiol treatment for 24 h. Empty vector was used as a control. qRT-PCR was performed to examine the expression level of miR-143. **B.**-**C.** T24 and 5637 cells were treated with siRNA against EZH2 (siEZH2) alone or combined with miR-143 inhibitor. Cell viability and clonogenicity were assessed by MTT assay **B.** and colony formation assay **C.**, respectively. **D.**, The expression levels of EZH2 and CD44 proteins in T24 and 5637 bladder cancer cells with EZH2 knockdown alone or combination with miR-143 inhibitor by Western blotting assay. The densitometry analysis was performed by Image J software and relative expression levels were indicated below blots. siCTL, nontargeting siRNA control; anti-miR NC, miRNA inhibitor negative control. *, *P* < 0.05; **, *P* < 0.01; ***, *P* < 0.001.

Whether miR-143 is the downstream target of EZH2 gene was investigated. It was found that knockdown EZH2 by siRNA in T24 and 5637 cells induced miR-143 expression greatly (Figure 4A and 4B); whereas ectopic expression of EZH2 in T24 cells significantly reduced miR-143 expression (Figure 4C and 4D). Furthermore, EZH2 overexpression represses the promoter activity of miR-143 by luciferase assay (Figure 4E). We then examined if honokiol regulates miR-143 expression through EZH2. Upon honokiol treatment, the decrease of EZH2 recruitment was detected in the promoter region (region 1), but not the downstream region (region 2, negative control; Figure 4F and 4G). Consistently, honokiol treatment only diminished the H3K27me3 level in region 1 on the promoter (Figure 4H). As shown in Figure 5A, ectopically expression of EZH2 into T24 cells significantly reversed the induction of miR-143 by honokiol. To further test whether miR-143 depletion can rescue cell growth arrest induced by EZH2 knockdown, we exploited miR-143 inhibitor to block miR-143 activity. In T24 and 5637 cells, miR-143 inhibitor increased the cell proliferation in MTT assay (Figure 5B) and elevated colony forming numbers (Figure 5C), respectively, comparing to the anti-miR negative control (CTRL inhibitor) under the same condition of EZH2 knockdown. In addition, the inhibition of miR-143 could re-induce the CD44 expression, which was suppressed by EZH2 knockdown in T24 and 5637 cells (Figure 5D). Altogether, honokiol down-regulates EZH2 expression, gets rid of the repressive H3K27me3 mark and induces miR-143 expression in human UBC cells.

### Honokiol inhibits T24 tumor growth in nude mice

**Figure 6:**
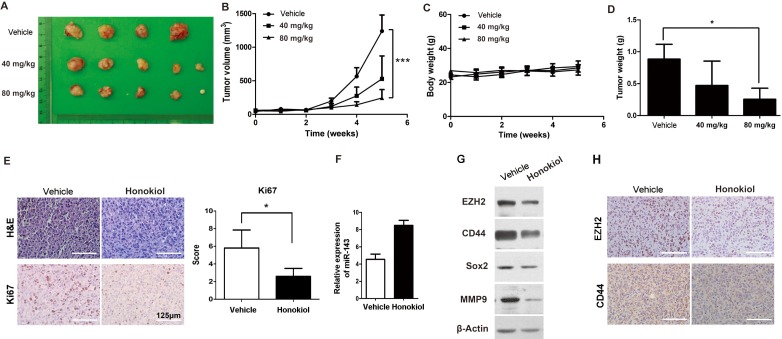
Honokiol decreased T24 UBC tumor growth *in vivo* A & B, photograph **A.** and tumor growths **B.** of vehicle, 40 mg/ml and 80 mg/ml honokiol groups. Measurements were performed every week for 5 weeks. **C.**, Body weight of T24 tumor-bearing mice upon honokiol treatment over time. **E.**, The average weights of tumors harvested from the vehicle, 40 mg/ml and 80 mg/ml honokiol-treated groups at the endpoint. **E.**, H&E and IHC staining of Ki67 on T24 tumor xenografts with vehicle or honokiol (80 mg/kg) treatment. **F.** & **G.**, The expression levels of miR-143 by qRT-PCR **F.**, and EZH2, CD44, Sox2, and MMP9 proteins by Western blot analysis **G.** in T24 tumor xenografts with vehicle or honokiol (80 mg/kg) treatment. **H.**, IHC staining of CD44 and EZH2 on T24 tumor xenografts with vehicle or honokiol (80 mg/kg) treatment.*, *P* < 0.05; ***, *P* < 0.001.

In order to evaluate the role of honokiol on tumor growth *in vivo*, T24 subcutaneous xenografts were generated in nude mice. When tumors became palpable, tumor-bearing animals were randomly divided into three groups for low-dose (40 mg/kg), high-dose (80 mg/kg) honokiol and vehicle treatments. Tumor size and animal body weight were measured every week for 5 weeks. Longitudinal studies of tumor volume and body weight in each cohort (n ≥ 4) were plotted over time. The tumor progression of high-dose honokiol group was significantly lower than that of vehicle group (Figure 6A and 6B, *P <* 0.001). There was no observed toxicity, for example, no significant body weight change (Figure 6C), even from the high-dose honokiol treatment in this study. Subcutaneous tumors with high-dose honokiol treatment were greatly smaller than control tumors. Average tumor weight at the endpoint in high-dose honokiol group (0.255 ± 0.174 g) was 28.8% of that in vehicle group (0.885 ± 0.232 g; Figure 6D, *P* < 0.05). The tumor proliferation rate was assessed by IHC staining for Ki-67. Ki-67-positive index was strikingly reduced in high-dose honokiol tumors comparing to the vehicle group (Figure 6E). Honokiol-induced upregulation of miR-143 was also observed (Figure 6F), consistent with the downregulation of EZH2 gene (Figure 6G and 6H). Protein levels of other stem cell markers, CD44 and Sox2, were also decreased in the T24 xenografts with honokiol treatment (Figure 6G and 6H).

## DISCUSSION

In this study, we are the first to report that honokiol suppresses EZH2/miR-143 axis to inhibit UBC cell proliferation, invasion, and stemness. The concentration of apoptosis induction effect of honokiol in our study is close to previous study using 50 μM honokiol (∼13.3 μg/ml) in T24 cells under different culture condition (0.5% serum) for 24 h [21]. In addition, we expanded the study to another two UBC cell lines, and found the anticancer effects of honokiol on UBC cells are general effect. Notably, our *in vivo* data implied that honokiol is a potential therapeutic agent for treating UBCs, with relatively minimal toxicity to the animals.

EZH2, a major histone methyltransferase in PRC2 complex, has been implicated in various cancer types, including prostate, lung and bladder cancer. Its overexpression is associated with cancer cell proliferation, cancer cell stemness and metastasis. For example, the elevated expression level of EZH2 is a common event in UBC samples, and correlates with aggressiveness and invasion of bladder tumors [22, 23]. In addition, EZH2 is also involved in the progression of prostate and breast cancer, which can serve as a marker of aggressive cancer types to predict clinical outcome [24]. Transgenic EZH2 promotes mammary tumor initiation of tumor virus-neu mice, through increasing the CSC population [25]. Additionally, EZH2 expression is required for pancreatic and breast cancer stem cell maintenance [26]. Hence, EZH2 is a promising therapeutic target for cancer. Our study demonstrated that EZH2 expression is enriched in the UBC stem cell population. Three UBC cell lines with various EZH2 expression levels show well response to honokiol treatment, suggesting that anti-cancer effects of honokiol is more general in UBC cells. Depletion of EZH2 by RNA interfering or honokiol treatment inhibited cell proliferation and clonogenicity. Of note, honokiol treatment significantly decreased tumor growth *in vivo* by downregulation of EZH2 expression and other aggressiveness related protein levels.

Since honokiol inhibits EZH2 at both mRNA and protein levels, such inhibition might be through transcription regulation. Recently, a couple of transcription factors and lncRNAs, such as NF-YA, hMOF and lncRNA-Sox2ot, have been implicated in EZH2 overexpression in ovarian, oral tongue squamous cell carcinoma and lung cancer [27-29]. How honokiol regulates EZH2 requires further investigation. Since ectopically expression of EZH2 in honokiol treated UBC cells can reverse the decreases of cell viability, colony formation capacity by honokiol, it suggests that EZH2 inhibition by honokiol is not a non-specific consequence of cell growth arrest. Overall, these data have demonstrated that inhibition of EZH2 by honokiol in UBC cells can suppress cancer cell growth and inhibit tumorigenicity.

The miRNAs play essential roles in various biological processes, including cell proliferation, survival, and differentiation. Downregulation of miR-143 was detected in many cancer types, including UBC [30-33]. In chronic ulcerative colitis, with high risk for neoplastic transformation, miR-143 was significantly increased, suggesting that loss of miR-143 might be an early event during colon cancer development [34]. Besides, the decrease of miR-143 expression in cancer cells induces cancer cell proliferation, migration, and chemoresistance [35-37]. Moreover, miR-143 has been reported to inhibit cancer cells stemness in glioblastoma stem-like cells and prostate cancer cells [38, 39]. Several genes, such as CD44, KLF5, K-Ras and hexokinase 2, have been confirmed as the direct targets of miR-143 in cancer cells [35, 40, 41]. Pagliuca A *et al*, has confirmed that miR-143 can reduced the expression level of CD44 (one stemness marker) through targeting CD44 3′UTR [35]. Altogether, it suggests that ectopic restoration of miR-143 mimic might be a promising approach for cancer treatment.

Multiple lines of evidence suggested that there is a complicated regulatory network controlled by the oncoprotein EZH2. EZH2 is directly recruited to target gene promoters and represses their transcriptions. These target genes include E-Cadherin, TIMP-3, and Slit2, which are responsible for cancer cell proliferation and invasion [42-44]. Recent studies revealed that microRNAs also play essential roles in cancer development, through targeting the 3′UTR of mRNAs. Only a small number of miRNAs, such as miR-200b, miR-200c and miR-31, have been found as the targets of EZH2 in cancer cells [16, 17]. Our data, for the first time, demonstrate that miR-143 is one of the direct targets of EZH2 in UBC cells. Transcriptional regulation mechanism of miR-143 has not been well-defined. Previous reports showed that DNA methyltransferase 3B may account for epigenetic regulation on the expression of miR-143 in endometrioid carcinomas [45]. In this study, we proved that EZH2 directly bound to the miR-143 promoter, modified the trimethylation status of histone H3K27 and repress the miR-143 transcription. Further functional analysis indicated that EZH2/miR-143 axis could confer accelerated cell proliferation and increased cell stemness in UBC cells both *in vitro* and *in vivo*.

The anticancer effects of honokiol might be through multiple pathways. Based on our findings, re-introduction of EZH2 or miR-143 inhibitor did not fully rescue honokiol-induced cell viability and colony formation capacities. These data implied that there must have some other regulators invovled in honokiol-induced cytotoxic effects. Recent studies showed that honokiol can target EGFR, AMPK, Notch signalings in various cancer cells; further supporting the idea that honokiol can regulate multiple pathways to inhibit cancer cells.

Taken together, our study suggested that honokiol could be a promising candidate for chemoprevention and/or therapeutic treatment. The inhibitory effect of honokiol on UBC cells may function through suppression of EZH2 function and induction of its target, miR-143.

## MATERIALS AND METHODS

### Reagents and antibodies

The animal protocol was approved by the Institutional Animal Care and Use Committee (IACUC), Model Animal Research Center of Nanjing University. Honokiol (HonoPure®, EcoNugenics, Santa Rosa, CA, USA) was dissolved in dimethyl sulfoxide (DMSO) and further diluted in sterile culture medium immediately prior to use. The final concentration of DMSO was less than 0.05%. Antibodies against H3K4me3, H3K9me3, H3K27me3, H3K79me3, H4K20me1, H4K20me2, Histone 3, Cyclin D1, CD44, EZH2, H3K27me3, p21, p27, PARP and β-Actin were purchased from Cell Signaling Technology (Danvers, MA, USA). Antibodies against Bcl2, Bax, MMP9, and Sox2 were purchased from Santa Cruz Biotechnologies (Santa Cruz, CA, USA). TRIzol RNA extraction reagent was purchased from Life Technologies (Carlsbad CA, USA). 20% Intralipid was purchased from Sigma (St. Louis, MO, USA).

### Cell culture

Human bladder cancer cell lines T24, 5637, J82, RT4, UMUC3, SW780, and BIU87 were obtained from the Cell Bank of Type Culture Collection, Chinese Academy of Science (Shanghai, China). Both cell lines were maintained in RPMI 1640 medium (Life Technologies), supplemented with 10% fetal bovine serum (FBS, Hyclone Laboratories, South Logan, UT, USA). Cell lines were incubated at 37°C in an atmosphere of 5% CO_2_ and 95% air.

### Cell proliferation assay

MTT (3-(4,5-dimethylthiazol-2-yl)-2,5-diphenyltetrazolium bromide) assay was performed to assess cell survival and growth. 1,500 cells/well were seeded to the 96-well plate in triplicate. Honokiol at various concentrations (4.8 μg/ml - 19.2 μg/ml) were added 24 h later. 3 days post treatment, cells were washed with PBS and MTT (5 mg/ml) was then added for 3 h at 37°C. 100 μl DMSO was loaded for each well to dissolve formazan crystals. Plates were shaken at room temperature for 15 min. Absorbance at 490 nm was examined using a microplate reader (BioTek Instruments, Winooski, VT, USA).

### Colony formation assay

500 cells per well were plated in 6-well plates. Two days later, different concentrations of honokiol (4.8 μg/ml - 19.2 μg/ml) were applied to UBC cells for 14 days. Cells were fixed with 100% methanol, washed with PBS and stained with 0.1% crystal violet. Only colonies with >50 cells were counted.

### Flow cytometry analysis

Cells were incubated with indicated concentrations of Honokiol for 48 h, before samples were fixed by 70% ethanol. Cells were incubated in 10 mg/ml RNase A containing PBS for 30 min at 37°C, followed by addition of 1 mg/ml propidium iodine (PI; Sigma). Afterwards cells were analyzed using a fluorescence-activated cell sorting (FACS) Calibur flow cytometer (BD FACS Calibur, BD Biosciences, San Jose, CA).

### Sphere formation assay

T24 and 5637 cells were trypsinized by TrypLE (Life Technologies Corp., Grand Island, NY, USA) and washed by PBS. 1.0×10^5^ cells per well were seeded in the 6-well ultra-low attachment plates (Corning, Steuben County, NY, USA) in DMEM/F-12 culture medium supplemented with 10 ng/ml human recombinant bFGF and 10 ng/ml EGF (PeproTech, Rocky Hill, NJ, USA). After culture for 14 days with or without honokiol, spheres were photographed. Spheres with the diameter greater than 50 μm were counted.

### Wound healing assay

Wound healing assay was used to assess the capacity of cell migration and invasion. Briefly, when the cells reached 90-95% confluence, the wound was generated by scratching the surface of the plates with a pipette tip. After removal of floating cells, adherent cells were incubated in various concentrations (4.8∼19.2 μg/ml) of honokiol or vehicle for 24 to 36 h till the wound was healed. The gap distances at different time points were normalized by the one immediately after scratching.

### Transwell invasion assay

The mixture of Matrigel (BD Biosciences, San Jose, CA, USA) and serum free cell culture medium (1:10) was added to upper chamber of 24-well transwell plates, incubated at 37°C 4 h for gelling, and kept at 4°C for 24 h. RPMI 1640 culture medium plus 10% FBS (500 μl/well) was added to the bottom well. 4×10^4^ cells in 100 μl were then seeded in the upper chamber with 8 μm pore membrane and incubated in honokiol at different concentration (4.8 μg/ml - 19.2 μg/ml) for 16 h. The cells on the membrane were fixed with 4% PFA for 10 min, stained with 0.1% crystal violet for 5 min and counted.

### Protein extraction and western blot analysis

Cells were lysed by ice-cold modified RIPA buffer. 20 μg of protein was resolved on SDS-PAGE gel, transferred to PVDF membrane (Millipore, Billerica, MA, USA). Primary antibodies against H3K4me3, H3K9me3, H3K27me3, H3K79me3, H4K20me1, H4K20me2, Histone 3, Cyclin D1, CD44, EZH2, MMP9, and Sox2 were used. β-Actin was used as a loading control. Chemiluminescence was achieved using the ECL Western Blotting Detection Reagents (Amersham Pharmacia Biotech, Arlington Heights, IL, USA).

### ALDEFLUOR assay

The ALDH enzyme activity was detected by the ALDEFLUOER kit (Stem Cell Technologies, Vancouver, Canada), according to the manufacturer's instructions. 5 μl of activated ALDEFLUOER reagent was added to 1 ml single cell suspension and incubated at 37°C for 40 min. An ALDH-specific inhibitor, Diethylaminobenzaldehyde (DEAB), was applied prior to staining as a negative control. Cells were analyzed on a BD FACScan flow cytometer (BD Biosciences).

### RNA isolation and quantitative RT-PCR (qRT-PCR)

Total RNA was extracted with TRIzol Reagent, according to the manufacturer's instructions. Small RNA (≤ 200 nucleotides) was extracted from tissue and cells by using mirVana miRNA isolation kit (Ambion), according to the manufacturer's instructions. Small RNA polyadenylation were performed according to the protocol as previously described [46]. Real-time PCR was performed in 384-well plates using SYBR Premix Ex Taq™ II kit (TaKaRa, Dalian, China) and measured by ABI 7900HT (Applied Biosystems, Carlsbad, CA, USA). *U6* (RNU6B) snRNA and *β-actin* (ACTB) mRNA were used as references for miRNA and mRNA, respectively. The primer sequences for qRT-PCR were listed in Supplementary Table S1.

### Plasmid construction

To clone miR143-promoter, we amplified 754-base pair (bp) promoter region from human genomic DNA by PCR using forward primer: 5′-CGGGGTACCGCAAAAGGAG AAAGAGGGACT-3′; reverse primer: 5′-CCCAAGCTTGAAGAGGCAAGCCCCGTATT-3′. And then we cloned the sequence into pGL3-basic (Promega) at KpnI and HindIII.

### Transfection

UBC cells were plated in 6 cm dish overnight, transfected with 20 μM of EZH2 siRNA (5′-GUGUAUGAGUUUAGAGUCAtt-3′) or scramble control siRNA (5′-UUCUCCGAACGUGUCACGUtt-3′), miR-143 mimic and miR-143 inhibitor (RIBOBIO, Guangzhou, China) by Lipofectamine 2000 (Life Technologies) for 48 h. For forced expression of EZH2, pcDNA3-EZH2 and empty vector (pcDNA3) were transfected into UBC cells by Lipofectamine 2000, respectively.

### Luciferase assay

Luciferase assays were performed using a luciferase assay kit (Promega) according to the manufacturer's instructions. Briefly, in 24-well plates T24 cells were transfected with pGL3-miR143-promoter plasmid with p3XFlag-CMV10 control vector (control group) and p3XFlag-CMV10-EZH2 (experiment group), respectively. The cells were harvested and lyzed for luciferase assay 48h after transfection. Renilla luciferase was used for normalization.

### Chromatin immunoprecipitation (ChIP) assay

ChIP Assay Kit (Millipore) was used to study the protein-DNA interaction in cells. Briefly, cross-linked DNAs were isolated from 5×10^6^ cells, followed by sonication into 300-1,000 bp in length. ChIP-grade antibodies against EZH2 (Cat# 5246, Cell Signaling) and trimethyl-H3K27 (Cat# 9733, Cell Signaling) were applied, respectively. Normal rabbit IgG antibody was used as a negative control. The sequences of primers used for qPCR analysis of the miR-143 promoter were listed in Supplementary Table S1.

### *In vivo* tumor xenograft study

2.5 × 10^6^ T24 cells in 0.1 ml 50% Matrigel were injected subcutaneously into the right flank of 4- to 6-week-old male athymic nude mice. 10 days after cell inoculation, the animals with palpable tumors were randomly divided into three experimental groups with intraperitoneal injections every other day for 5 weeks: (1) low dose honokiol, 40 mg/kg in 20% Intralipid; (2) high dose honokiol, 80 mg/kg in 20% Intralipid; and (3) vehicle control, 20% Intralipid. Tumor volume was calculated by the formula tumor volume [mm^3^] = (length [mm]) × (width [mm])^2^/2. At the endpoint, tumors were weighed and processed for further histological analysis.

### Immunohistochemistry (IHC)

5 μm-thick paraffin sections were for IHC staining. The primary antibodies against CD44 (Cell Signaling, #3570, 1:200) and Ki67 (Vector Laboratories, VP-RM04, 1:300) were incubated overnight at 4°C, respectively. Secondary antibody conjugated with horseradish peroxidase (HRP, KIT-9701, Maxim Biotechnologies, China) was then incubated for 1 h at room temperature. The slides were developed with the NovaRED substrate kit (Vector Laboratories, SK-4800) and counterstained by hematoxylin. A score was assigned to represent the estimated percentage of positively stained carcinoma cells as follows: 0: none, 1: ≤50%, 2: 50-75%, and 3: ≥75%. An intensity score was assigned to represent the average estimated intensity of staining in positive carcinoma cells as follows: 0: none, 1: weak, 2: intermediate, 3: strong. The proportion score and intensity score were multiplied to obtain a total score ranging from 0 to 9.

### Statistical analysis

Each experiment was repeated three times. The mean values with standard deviations from the triplicates were plotted. The significant difference between control and experimental groups was analyzed using *t*-test (* *P* < 0.05; ** *P* < 0.01; *** *P* < 0.001).

## SUPPLEMENTARY MATERIAL FIGURES AND TABLE



## Abbreviations

UBC: urinary bladder cancer
CSC: cancer stem cell
miRNA: microRNA
PRC: polycomb repressive complex
EZH2: Enhancer of Zeste homologue 2
MTT: 3-(4,5-dimethylthiazol-2-yl)-2,5-diphenyltetrazolium bromide
DMSO: dimethyl sulfoxide
qRT-PCR: quantitative reverse transcription-PCR
bp: base pair
