# Supplementary material for: Honokiol inhibits bladder tumor growth by suppressing EZH2/miR-143 axis
Source: Oncotarget. 2015 Oct 15;6(35):37335–48. doi: 10.18632/oncotarget.6135 (PMC4741933; doi:10.18632/oncotarget.6135)
Supplement: Supplementary file 1 [file oncotarget-06-37335-s001.pdf]

Honokiol inhibits bladder tumor growth by suppressing EZH2/miR-143 axis

Supplementary Material

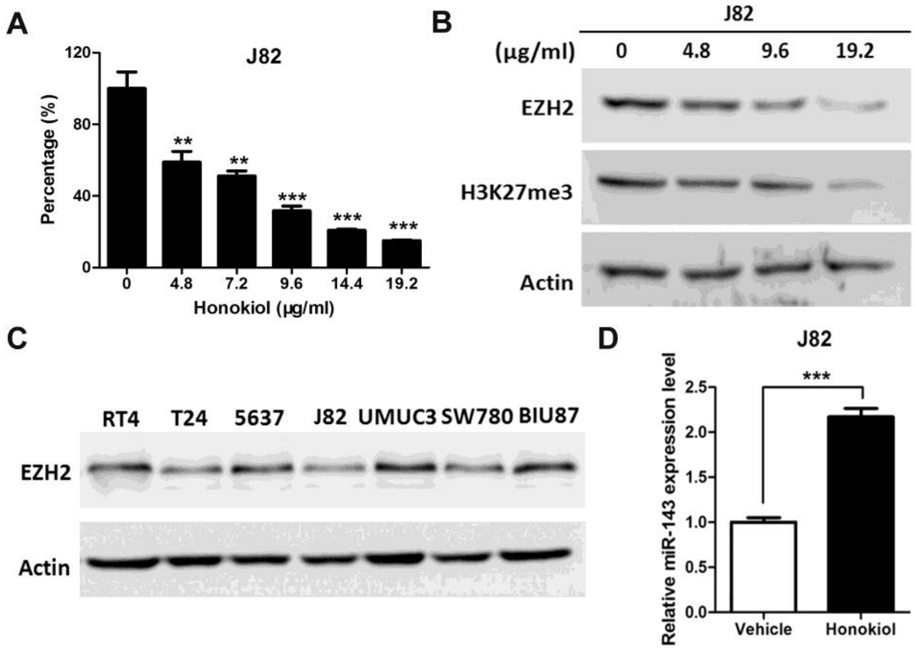

Supplementary Figure 1. Anti-cancer effects of Honokiol on J82 human UBC cells. (A) Cytotoxic effects of honokiol on J82 cells for 24 h. (B) The changes of EZH2 and H3K27me3 levels in honokiol treated J82 cells for 24 h by Western blotting. (C) The expression level of EZH2 in multiple human UBC cell lines. (D) The induction of miR-143 by 9.6 μg/ml honokiol for 24 h in J82 cells. **, *P* < 0.01; ***, *P* < 0.001.

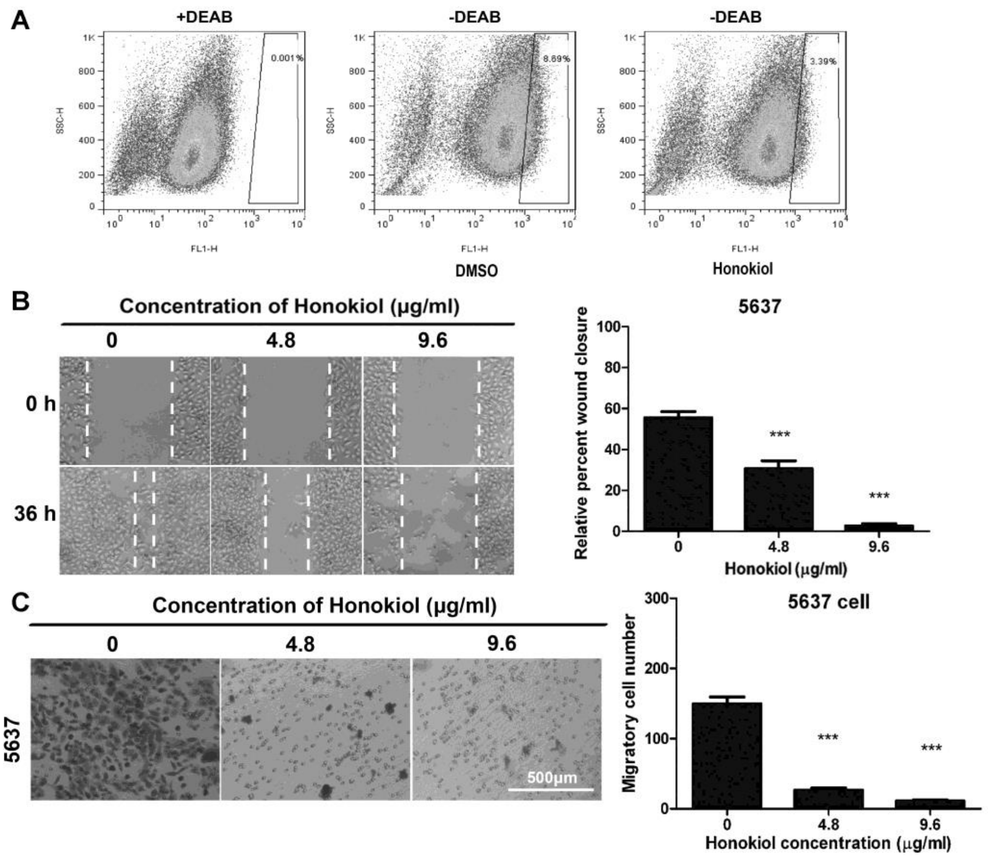

Supplementary Figure 2. Honokiol suppresses cancer cell stemness, migration, and invasion of 5637 cells. A, ALDH^{High} cell population (stem and progenitor cells) assessed by flow cytometric analysis in 5637 cells treated with 9.6 µg/ml honokiol for 24 h. Diethylaminobenzaldehyde (DEAB), an ALDH-specific inhibitor, was used as a negative control. B, Cell migration capacity examined by the wound healing assay. Relative wound closure percentage at 24 h was normalized by the gap distance at 0 h. Vertical dotted lines indicate the wound formed at 0 h (top row) and 24 h (bottom row). C, Cell invasive capacity was detected by the Transwell assay. Representative photograph of the transwell inserts with invasive cells at 20× magnification. ***, $P < 0.001$, compared with the vehicle-treated group indicated as “0”.

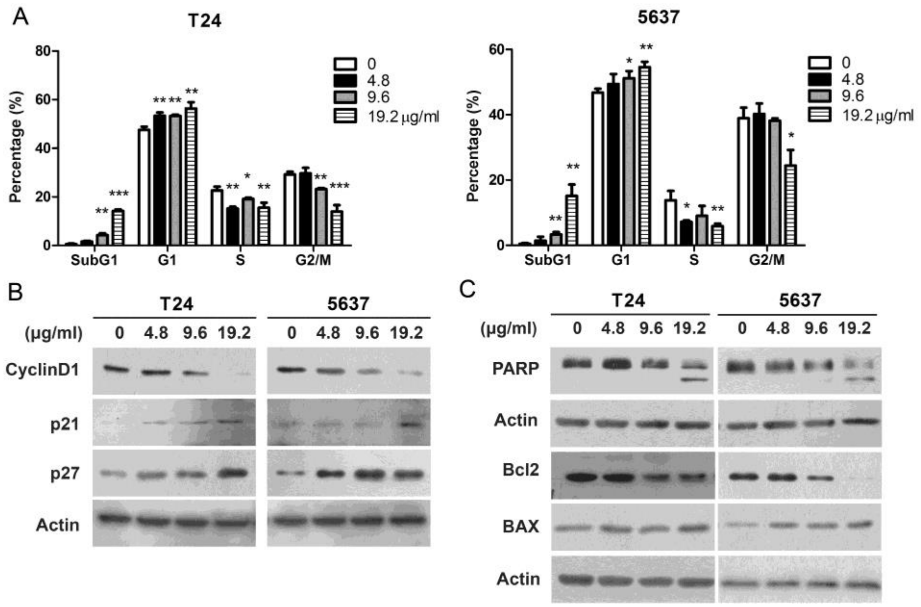

Supplementary Figure 3. Honokiol induced G1 arrest and apoptosis in UBC cells. (A) Flow cytometry analysis of T24 and 5637 cells treated with honokiol with the indicated concentrations for 24 h. (B) Western blotting of G1 arrest by honokiol. Cell proliferation marker (Cyclin D1) and cell cycle inhibitors (p21 and p27) were analyzed. (C) Western blotting of apoptosis related proteins by honokiol treatment. The pro-apoptotic protein (BAX) and anti-apoptotic protein (Bcl2) with the apoptotic marker (cleavages of PARP) were detected. *, $P < 0.05$, **, $P < 0.01$, ***, $P < 0.001$.

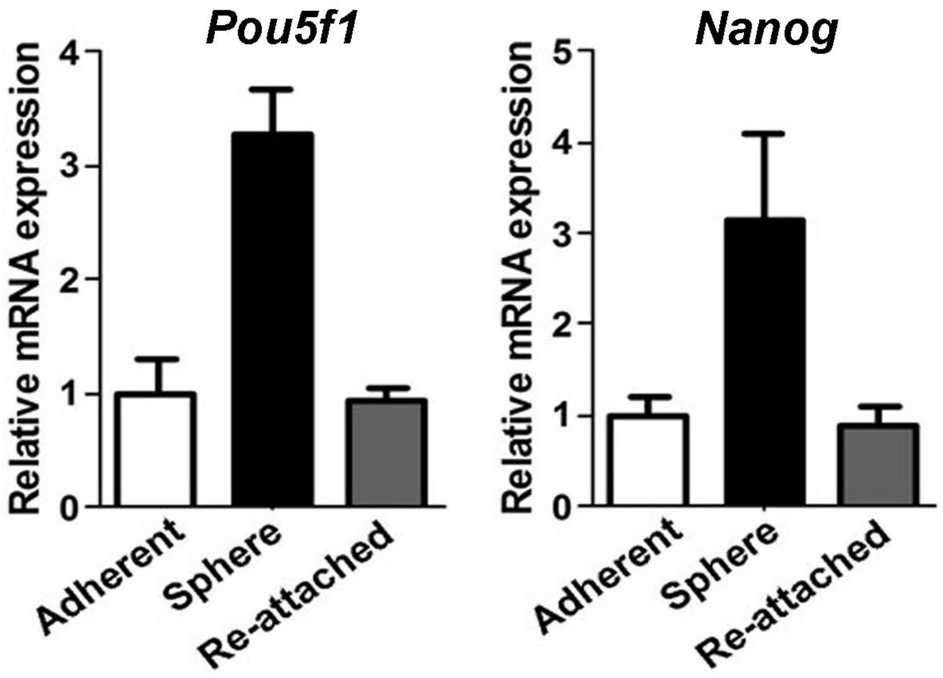

Supplementary Figure 4. Expression of *Pou5f1* and *Nanog* in stem/progenitor cell population of T24 cells. *Pou5f1* and *Nanog* mRNA levels in T24 tumor sphere (stem/progenitor cell population) and T24 cells re-attached on cell plates (differentiated cell population) by quantitative RT-PCR. *Pou5f1* and *Nanog* mRNA levels in T24 parental cells under regular culture condition (adherent) was used as the normalization control.

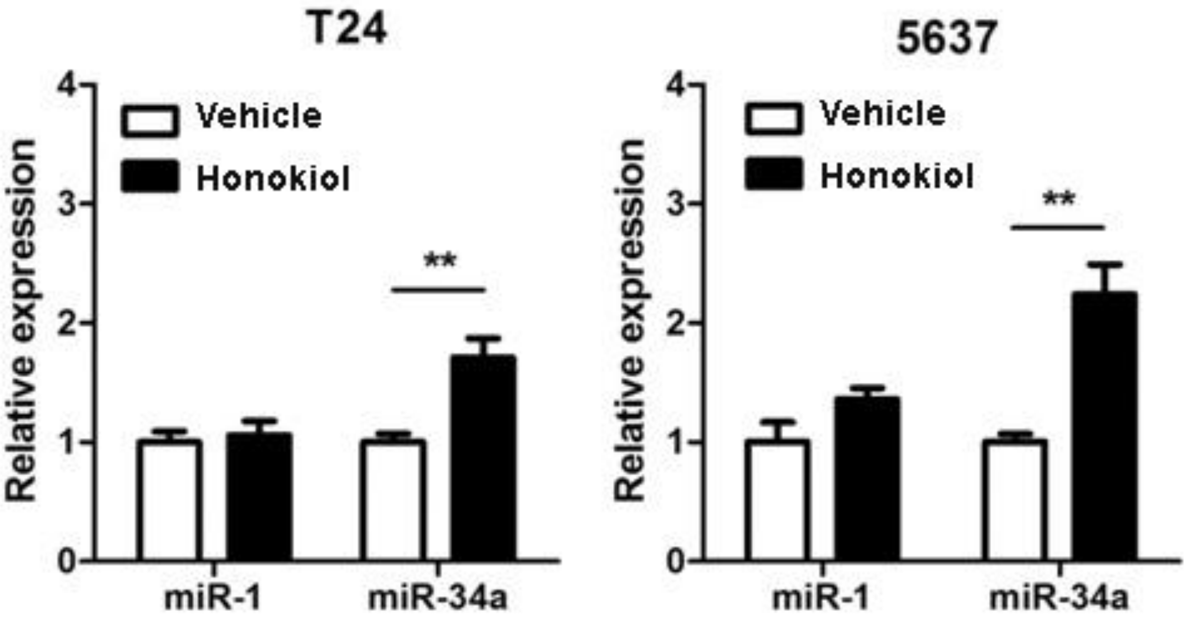

Supplementary Figure 5. Expression changes of miR-1 and miR-34a upon treatment of honokiol. The expression levels of negative control (miR-1) and positive control (miR-34a) were examined in 9.6 μg/ml honokiol-treated T24 and 5637 cells for 24 h. **, $P < 0.01$.

Supplementary Table 1. Sequences of primers for qRT-PCR and ChIP assays

Genes	Direction	Primer sequences
**qRT-PCR for mRNA**		
EZH2	Forward	5’-GGACTCAGAAGGCAGTGGAG-3’
	Reverse	5’-GCCAACAAACTGGTCCCTT-3’
Aldh1a1	Forward	5’-ATTGCTGAGCCAGTCACCTG-3’
	Reverse	5’-GCCTTGTCAACATCCTCCTTATCT-3’
Pou5f1	Forward	5’-CAAAGCAGAAACCCTCGTGC-3’
	Reverse	5’-CTCGGACCACATCCTTCTCG-3’
Nanog	Forward	5’-AAGGTCCCGGTCAAGAAACAG-3’
	Reverse	5’-CTTCTGCGTCACACCATTGC-3’
CD44	Forward	5’-AGCAACTGAGACAGCAACCA-3’
	Reverse	5’-AGACGTACCAGCCATTTGTGT-3’
Sox2	Forward	5’-TTTGTCGGAGACGGAGAAGC-3’
	Reverse	5’- TAACTGTCCATGCGCTGGTT-3’
**qRT-PCR for human miRNA**		
Uni-primer		5’-CGAATTCTAGAGCTCGAGGCAGG-3’
U6		5’-ATTCGTGAAGCGTTCCATAT-3’
let-7a		5’-TGAGGTAGTAGGTTGTATAGTT-3’
miR-1		5’-TGGAATGTAAAGAAGTATGTAT-3’
miR-101a		5’-TACAGTACTGTGATAACTGAA-3’
miR-122		5’-TGGAGTGTGACAATGGTGTT-3’
miR-133		5’-TTTGGTCCCCTTCAACCAGC-3’
miR-143		5’-TGAGATGAAGCACTGTAGCTC-3’
miR-145a		5’-GTCCAGTTTTCCCAGGAATCCCT-3’
miR-155		5’-TTAATGCTAATCGTGATAGGGGT-3’
miR-16		5’-TAGCAGCACGTAAATATTGGCG-3’
miR-182		5’-GGCAATGGTAGAACTCACACCG-3’
miR-183		5’-TATGGCACTGGTAGAATTCACT-3’
miR-200b		5’-TAATACTGCCTGGTAATGATGA-3’
miR-200c		5’-TAATACTGCCGGGTAATGATGGA-3’
miR-206		5’-TGGAATGTAAGGAAGTGTGTGG-3’
miR-208b		5’-ATAAGGACGAGCAAAAAGCTTGT-3’
miR-210		5’-CTGTGCGTGTGACAGCGGCT-3’
miR-223		5’-TGTCAGTTTGTCAAATACCCCA-3’
miR-34a		5’-TGGCAGTGTCTTAGCTGGTTGT -3’
miR-378		5’-ACTGGACTTGGAGTCAGAAG-3’
*miR-378**		5’-CTCCTGACTCCAGGTCCTGT-3’
miR-499		5’-TTAAGACTTGCAGTGATGTT-3’
miR-9		5’-TCTTTGGTTATCTAGCTGTATGA-3’
miR-96		5’-TTTGGCACTAGCACATTTTTGC-3’
**ChIP assay**		
Region 1	Forward	5’-TACTCCCATCATCATGCAAA-3’

	Reverse	5’-GAAGGACATTCCACATTCAA-3
Region 2	Forward	5’-CAGAACTGTGAGTCAATTAA-3’
	Reverse	5’-ATCCTCCAAACTGCCAT ATT-3’
